# [Retracted] Inhibition of RKIP aggravates thioacetamide‑induced acute liver failure in mice

**DOI:** 10.3892/etm.2023.12005

**Published:** 2023-05-10

**Authors:** Xing Lin, Jinbin Wei, Jinlan Nie, Facheng Bai, Xunshuai Zhu, Lang Zhuo, Zhongpeng Lu, Quanfang Huang

Exp Ther Med 16:2992–2998, 2018; DOI: 10.3892/etm.2018.6542

Following the publication of the above paper, an interested reader drew to our attention that a number of apparent anomalies existed with the data presented in the above paper. First, there appeared to be strikingly similar and duplicated patternings of the cells within the cellular images featured in Fig. 2, images 3 and 4 (showing the data for the TAA model and TL groups, respectively). Secondly, certain of the data panels shown in Fig. 4 appeared to be strikingly similar to data shown in Fig. 4 in a previously published paper, which featured several of the same authors [Lin X, Liu X, Huang Q, Zhang S, Zheng L, Wei L, He M, Jiao Y, Huang J, Fu S *et al*: Hepatoprotective effects of the polysaccharide isolated from *Tarphochlamys affinis* (Acanthaceae) against CCl4-induced hepatic injury. Biol Pharm Bull 35: 1574-1580, 2012]. Finally, it was pointed out that some of the western blot images presented in Fig. 4B in the above paper had also apparently been featured in different formats in two other publications that also contained several of the same authors.

Following an internal enquiry, the Editor of *Experimental and Therapeutic Medicine* has been able to verify the claims made by the interested reader; therefore, in view of the number of potential anomalies that have been identified and owing to a lack of overall confidence in the presented data, the Editorial Board have decided to retract the above paper from the publication. The authors were asked for an explanation to account for these concerns, but the Editorial Office did not receive a satisfactory reply. The Editor apologizes to the readership of the Journal for any inconvenience caused.

